# Editorial: The advances in proteomics and targeted therapy of malignant brain tumours

**DOI:** 10.3389/fonc.2023.1195847

**Published:** 2023-04-25

**Authors:** Anjing Chen, Zhenyi An

**Affiliations:** ^1^ Department of Neurosurgery, Qilu Hospital, Cheeloo College of Medicine and Institute of Brain and Brain-Inspired Science, Shandong University, Jinan, China; ^2^ Jinan Microecological Biomedicine Shandong Laboratory, Shandong Key Laboratory of Brain Function Remodeling, Jinan, China; ^3^ Broad Institute of Massachusetts Institute of Technology (MIT) and Harvard, Cambridge, MA, United States

**Keywords:** malignant brain tumours, proteomics, targeted therapy, advances, glioma

Malignant brain tumours are rapidly growing cancers that are often life-threatening due to their invasion of surrounding healthy brain structures. Malignant brain tumours account for around a third of all primary brain tumours, with high-grade gliomas the most common type. Despite progress in treatment including surgery, radiation, and chemotherapy, the prognosis for patients with malignant brain tumours remains dismal. Heterogeneity at genomic, transcriptomic, proteomic, and metabolomic levels among patients emphasizes the necessity of developing personalized and targeted therapy. Precision medicine for malignant brain tumours faces challenges in the identification of biomarkers, systematic description of pathogenic mechanisms, and accurate evaluation of therapeutic efficacy. Recent advancement of large-scale omics technologies, especially proteomics studies, has greatly promoted the discovery of new biomarkers. Follow-up research, understanding how the key biomarker proteins work to regulate tumorigenesis, the tumor microenvironment, and tumor growth and stemness is vital for the development of new targeted therapies that can eventually translate to patients. We collected seven interesting articles including four original research articles and three review articles.

Two original research articles by Rana et al. and Sitter et al., show Cytochrome C as a potential biomarker for glioma and reveal multiple variables associated with serum lactate in glioma, respectively. The article by Rana et al. focuses on identifying glioma biomarkers in apoptosis and related pathways. They evaluated the expression levels of apoptotic proteins in healthy controls (n=6) and different grades of gliomas (grade I-IV, n=6 for each grade) using an apoptosis protein array. Five proteins in the array, Clusterin, HSP27, Catalase, Cytochrome C, and SMAC, showed significant changes in protein levels from low to high-grade glioma. While Cytochrome C and SMAC levels decreased with higher grades, Clusterin, HSP27 and Catalase levels increased with higher grades. The authors further confirm the finding of cytochrome C in glioma patient samples with different grades using immunofluorescent staining and flow cytometry. The authors also list a group of drugs that could target Cytochrome C and related pathways, providing some clues for follow-up drug development. In the article by Sitter et al., the authors perform a clinical study in 261 glioma patients to evaluate the correlation between serum lactate and a group of variables including age, tumor volume, edema, and maximum tumor diameter. High serum lactate levels showed a weak correlation (coefficient between 0.1 and 0.39) with higher blood glucose, larger tumor volumes, and more tumor edema, and a moderate correlation with corticosteroid treatment (coefficient 0.477). As corticosteroid treatment tends to be a common trait in glioma, the authors reason that the potential for using serum lactate as a biomarker in glioma could be low.

Two original research articles from Ohba et al. and Chen et al., show the molecular mechanism of pyruvate kinase M2 (PKM2) and C2H2 Zinc finger 65 (ZNF655) in the regulation of malignant progression of glioma, respectively. The article by Ohba et al. shows that the knockdown of PKM2 results in an accumulation of cells in G2-M phase and decreased Cdk1 activity. PKM2 bound and stabilized the Cdk1-cyclinB complex through Y15 phospho-Cdk1, facilitating Cdk1-cyclin B activation and cell cycle progression. The results identify PKM2 as an integral component of the Cdk1-cyclinB complex and indicate that therapeutics altering this interaction could be considered in the future to inhibit tumor cell growth. Chen et al. study the biological function and clinical significance of ZNF655 in glioma. They find that ZNF655 is abundantly expressed both in glioma tissues and cultured glioma cell lines. Knockdown of ZNF655 in glioma cells decreases proliferation, enhances apoptosis, inhibits migration, and weakens tumorigenesis. Mechanism studies indicate that ZNF655 activates the expression of AURKA by binding to the promoter of AURKA. Knockdown of AURKA alleviates the effects of ZNF655 overexpression. This study indicates that AURKA/ZNF655 could be candidates to target in glioma.

Three review articles focus on discussing small molecule inhibitors, epithelial-mesenchymal transition (EMT) of glioma, and the application of proteomics in the discovery of radiosensitive cancer biomarkers. The review article by Huang et al. discusses the history and future of small molecule inhibitors in adult high-grade glioma. The study summarizes the dysfunctional molecular mechanisms and highlights the outcomes of relevant clinical trials associated with small molecule targeted therapies. Another review article, by Xing et al., provides a scientometric analysis of emerging trends and research foci of epithelial-mesenchymal transition (EMT) in gliomas using a total of 1217 publications. The authors summarize studies focusing on the cellular and molecular mechanisms of EMT and therapies related to EMT target and noncoding RNAs in gliomas. This review also discusses currently known mechanisms of EMT, pointing the direction of the development of new therapies for gliomas. A mini-review by Luo and Ge discusses the application of proteomics in the discovery of radiosensitive cancer biomarkers. The authors introduce five broadly-used mass spectrometry technologies including MALDI-TOF-MS, iTRAQ, LC-MS/MS, MRM and SWATH-MS, and highlight their application in identifying new radiosensitive markers in head and neck cancers, thoracic cancers, abdominopelvic tumours and extracranial tumours in children. The authors also discuss the remaining challenges of proteomics and future directions in the discovery of radiosensitive cancer biomarkers using proteomics.

Altogether, this Research Topic emphasizes the identification and characterization of biomarkers in malignant brain tumours. We highlight proteomic-related technologies in biomarker discovery and studies elucidating the working mechanisms of different biomarkers. These results cover the fields of tumor precision molecular typing, personalized therapy, and drug development. A combination of basic research, clinical research, and review articles are included to provide a broad view of the advances in these fields. The collected findings of this Research Topic suggest new avenues for precision medicine in malignant brain tumours and highlight the important roles of proteomics technologies.

## Author contributions

Writing, Review, and Editing: AC and ZA. All authors contributed to the article and approved the submitted version.

